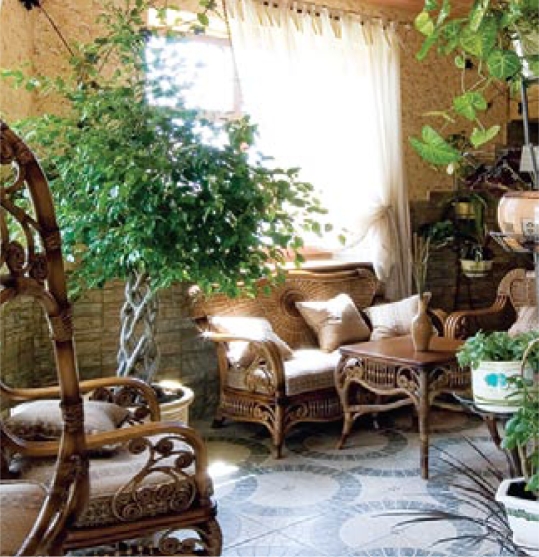# The Beat

**Published:** 2009-10

**Authors:** Erin E. Dooley

## Vehicles Concentrate Nicotine

A study by Patrick Breysse et al. published online 25 August 2009 ahead of print in *Tobacco Control* found that vehicle passengers riding with smokers may be exposed to nicotine levels 40–50% higher than those found in restaurants and bars that permit smoking. Nicotine concentrations inside vehicles increased twofold for every cigarette smoked, and while opening the windows reduced smoke somewhat, it did not eliminate exposure within the vehicle. Breysse et al. state the levels are unacceptable for nonsmoking passengers, especially children, who are at increased risk for secondhand smoke–related health problems..

**Figure:**
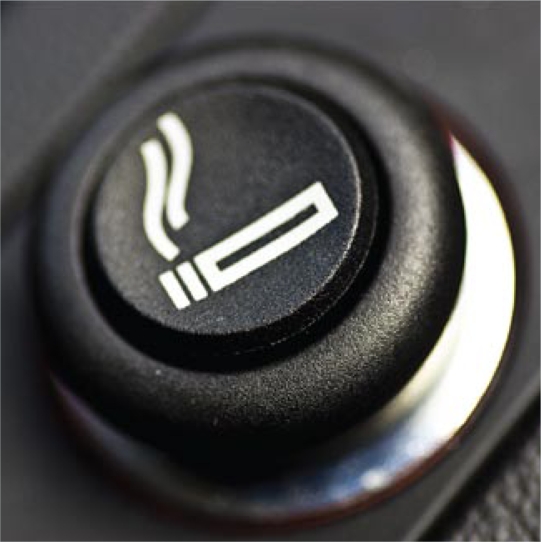


## USDA Maps Local Food Production

Until recently, low fuel prices meant the U.S. food system could rely on food imported from other countries, but rising transport costs and regional food shortages and crop failures are among several factors encouraging a closer look at local food production. Wayne Honeycutt and colleagues at the Agricultural Research Service are now mapping data from Maine to Virginia on weather, soil, land use, and water availability to model potential crop production and determine local food production capacity. They say expanding opportunities for local food production could stimulate rural development and offset the risk of food shortages by diversifying and increasing local production in other areas.

**Figure:**
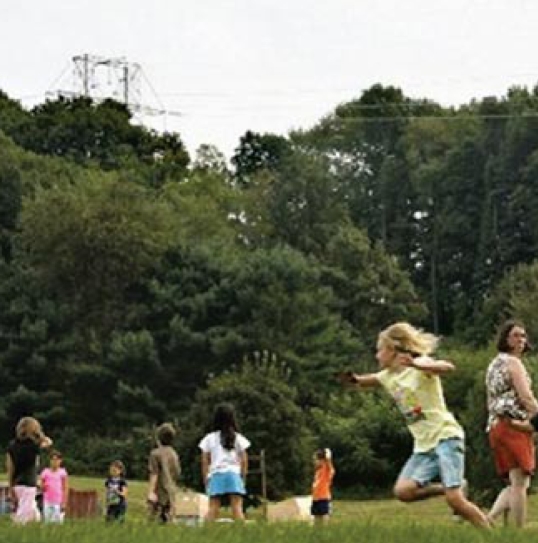
Fredon Township School playground

## School Averts EMF-Related Closure

The debate over the safety of electromagnetic fields (EMFs) nearly closed a New Jersey school this fall until the state’s biggest electric utility and the Sussex County Board of Education reached an eleventh-hour settlement. The board had planned to close the K–6 Fredon Township School 1 October 2009 because the existing high-voltage power line crossing the school’s playground had been found to emit EMF levels more than 8 times the WHO-recommended maximum of 3 milligauss. Under the settlement, Public Service Electric & Gas Co. agreed to pay $95,000 to relocate playgrounds located under its lines. There is limited evidence that EMFs from power lines may be a risk factor for childhood leukemia.

## Colorimetric “Nose” Alerts Chemical Handlers

In a report published online 13 September 2009 in *Nature Chemistry*, Kenneth Suslick and colleagues present a postage stamp–sized electronic sensor capable of quickly and inexpensively detecting toxic chemicals and their concentrations through color visualization. The pattern created by color changes in the disposable 36-dye sensor array identifies both the toxicant and its concentration. The sensor can detect more chemicals than previous methods and produces most results within 2 minutes. The researchers have developed a handheld version similar to a card-scanning device that uses LED illumination and an ordinary camera.

## Take-Home Dust Boosts Car Seat Lead Levels

Several studies have established that lead-exposed workers can carry lead-contaminated dust off the jobsite on their clothing, shoes, and tools. In the 21 August 2009 *MMWR* Tina Bernier et al. report the first known cases of childhood lead poisoning attributed to take-home lead dust deposited onto car safety seats. No contamination was found in the six children’s homes, leading the researchers to examine family vehicles and car seats, where high lead levels were found. Although previous studies have recommended monitoring blood lead levels among children of lead-exposed workers, no standards exist for levels of lead dust contamination in vehicles or on child car safety seats.

## Indoor Greenery Releases VOCs

Peace lilies, snake plants, weeping figs (ficus trees), and areca palms are just a few of the houseplants that have been shown to remove volatile organic compounds (VOCs) from indoor air, but a new study by Dong Sik Yang et al. in the August 2009 issue of *HortScience* finds they can also release these chemicals. The authors found these four types of houseplants released 12–23 VOCs, and although the researchers did not quantify potential exposures, they did note that emissions were higher during the day than at night. The authors attributed the VOCs to pesticides used in nurseries, microorganisms living in the growing medium, and offgassing of plastic planters.

**Figure:**